# Carriage of ESBL-producing *Klebsiella pneumoniae* and *Escherichia coli* among children in rural Ghana: a cross-sectional study

**DOI:** 10.1186/s13756-023-01263-7

**Published:** 2023-07-03

**Authors:** Charity Wiafe Akenten, Neyaz Ahmed Khan, Joyce Mbwana, Ralf Krumkamp, Dennis Fosu, Ellis Kobina Paintsil, Kennedy Gyau Boahen, James Osei-Mensa, Oumou Maiga-Ascofare, Jürgen May, Kwasi Obiri-Danso, Richard Odame Phillips, Linda Aurelia Ofori, Denise Dekker

**Affiliations:** 1 Kumasi Centre for Collaborative Research in Tropical Medicine, Kumasi, Ghana; 2 Infectious Disease Epidemiology Department, Bernhard Nocht Institute for Tropical Medicine, Hamburg, 20359 Germany; 3 One Health Bacteriology Group, Bernhard Nocht Institute for Tropical Medicine, Hamburg, 20359 Germany; 4 National Institute for Medical Research, Tanga, Tanzania; 5 German Centre for Infection Research (DZIF), Hamburg-Lübeck-Borstel-Riems, Germany; 6 University Medical Centre Hamburg-Eppendorf (UKE), Tropical Medicine, Hamburg, Germany; 7 Kwame Nkrumah University of Science and Technology, Kumasi, Ghana

**Keywords:** Extended-spectrum beta-lactamase, Carriage, Diarrhoea, Non-diarrhoea, *Klebsiella pneumoniae*, *Escherichia coli*, Children, Ghana

## Abstract

**Background:**

Extended-spectrum beta-lactamase (ESBL)-producing *Klebsiella pneumoniae* (ESBL-KP) and *Escherichia coli* (ESBL-EC) present a high burden in both communities and healthcare sectors, leading to difficult-to-treat infections. Data on intestinal carriage of ESBL-KP and ESBL-EC in children is scarce, especially in sub-Saharan African countries. We provide data on faecal carriage, phenotypic resistance patterns, and gene variation of ESBL-EC and ESBL-KP among children in the Agogo region of Ghana.

**Methods:**

From July to December 2019, fresh stool samples were collected within 24 h from children < 5 years with and without diarrhoea attending the study hospital. The samples were screened for ESBL-EC and ESBL-KP on ESBL agar and confirmed using double-disk synergy testing. Bacterial identification and an antibiotic susceptibility profile were performed using the Vitek 2 compact system (bioMérieux, Inc.). ESBL genes, *bla*SHV, *bla*CTX-M, and *bla*TEM were identified by PCR and further sequencing.

**Results:**

Of the 435 children recruited, stool carriage of ESBL-EC and ESBL-KP was 40.9% (n/N = 178/435) with no significant difference in prevalence between children with diarrhoea and non-diarrhoea. No association between ESBL carriage and the age of the children was found. All isolates were resistant to ampicillin and susceptible to meropenem and imipenem. Both ESBL-EC and ESBL-KP isolates showed over 70% resistance to tetracycline and sulfamethoxazole-trimethoprim. Multidrug resistance was observed in over 70% in both ESBL-EC and ESBL-KP isolates. The *bla*CTX-M-15 was the most prevalent ESBL gene detected. *bla*CTX-M-27, *bla*CTX-M-14, and *bla*CTX-M-14b were found in non-diarrhoea stools of children, whereas *bla*CTX-M-28 was found in both the diarrhoea and non-diarrhoea patient groups.

**Conclusions:**

The carriage of ESBL-EC and ESBL-KP among children with and without diarrhoea in the Agogo community with a high prevalence of *bla*CTX-M-15 is noteworthy, highlighting the importance of both the population as a possible reservoir. This study reports for the first time the ESBL gene *bla*CTX-M-28 among the studied populations in Ghana.

## Introduction

Antimicrobial resistance (AMR) is one of the top ten global health threats to humans [1]. In 2019, AMR was directly responsible for 1.2 million deaths and was associated with an estimated 4.6 million deaths worldwide [2]. AMR, due to extended-spectrum beta-lactamase (ESBL)- producing bacteria, has escalated over the past years, both in hospitals and in communities [3]. Worldwide, the rapid spread of resistant pathogens has been mainly attributed to the overuse of antibiotics in human medicine and animal husbandry in sub-Saharan African (SSA) regions [4]. Currently, many Gram-negative bacteria can produce ESBL enzymes, conferring resistance to penicillins, first-, second-, and third-generation cephalosporins, and aztreonam (but not carbapenems or cephamycins) [3]. ESBL genes, which were first reported among *Klebsiella* spp. and *Escherichia coli*, are rapidly spreading among other bacteria through plasmid-mediated horizontal gene transfer [5]. In addition, ESBL-encoding plasmids can also code for other non-beta-lactam resistance genes, leading to multi-drug resistance [6]. In SSA, infections caused by ESBL-producing bacteria, including *E. coli* (ESBL-EC) and *Klebsiella pneumoniae* (ESBL-KP), are of great concern. Most *E. coli* and *K. pneumoniae* are causative agents of infections such as bacteraemia, urinary tract infections, and diarrhoea, particularly among children, both in hospital and community settings [7, 8].

Admittedly, *E. coli* and *K. pneumoniae* isolated from the stools of patients are not typically associated with diarrhoea except for diarrheagenic *E. coli* such as STEC and EHEC [9]. However, other gastrointestinal bacteria, for example, *Salmonella enterica*, are associated with severe diarrhoea. In case such pathogens are ESBL producers, they can cause difficult-to-treat infections leading to life-threatening complications. Recent studies have highlighted faecal carriage as a significant reservoir of ESBL-producing bacteria in hospitals and communities [10, 11]. The colonization of the intestinal tract with ESBL-carrying bacteria has been shown to precede infections [12], and hence stool carriage of ESBL-EC and ESBL-KP is of medical importance. In SSA, studies have reported carriage of ESBL-EC and ESBL-KP in the intestinal tract of humans and animals, highlighting that also individuals without gastrointestinal symptoms can be carriers of ESBL [13–16].

This study is of descriptive nature, aiming to determine the prevalence, antibiotic resistance, and gene variation of ESBL-EC and ESBL-KP in children with and without diarrhoea attending a rural hospital and child clinic in Agogo, Ghana.

## Materials and methods

### Study site and study population

A cross-sectional study was conducted to determine the frequency, antibiotic resistance, and gene variation of ESBL-EC and ESBL-KP in children with and without diarrhoea. The study was conducted at the Agogo Presbyterian Hospital (APH) and a selected Child Welfare Clinic (CWC) where parents and guardians take their children under five years of age for routine check-ups in the Agogo community in the Asante Akyem municipal district, Ashanti region of Ghana. Between June and December 2019, children below five years of age living in Agogo and nearby communities were recruited and categorized into either of the following two groups: (1) children with diarrhoea or episodes of diarrhoea within the last 72 h; and (2) children without diarrhoea and symptom free, attending CWC for routine immunization and growth monitoring with no history of diarrhoea for at least one month before study enrolment.

### Ethical approval

The study was approved by the Committee on Human Research, Publications, and Ethics at the School of Medical Science, Kwame Nkrumah University of Science and Technology, Kumasi, Ghana, and by the German Medical Association (CHRPE/AP/593/17 and (CHRPE/AP/119/22). All participants were informed about the purpose of the study. Written informed consent was obtained from the parent or guardian of each child before study enrolment.

### Sample collection

Stool samples were collected in sterile containers. In cases where stool samples were not readily available, a team member followed up with the parent or guardian to obtain a stool sample within 24 h. All stool samples were transported in a cool box at 2–8 °C to the laboratory of the Kumasi Center for Collaborative Research in Tropical Medicine (KCCR) for analysis within 4 h of taking the sample. To monitor the temperature, one pack of ice cool aid was put in a Va-Q-bagi (Bereg-Kit, Switzerland) with a thermometer.

### Identification of E. coli and K. pneumoniae isolates and their antimicrobial susceptibility profiles

Stool samples were cultured on two MacConkey agar plates supplemented with 1 mg/L ceftazidime and 1 mg/L cefotaxime, respectively. Plates were incubated at 35–37°C for 18–24 hours in a normal atmosphere. Lactose-fermenting colonies (not more than three colonies), with typical morphology presumptive of *E*. *coli* and *K. pneumoniae*, were selected and sub-cultured on blood agar (Columbia Agar supplemented with 5% sheep blood) for isolation of pure colonies. The VITEK 2 Compact system, using Gram-negative bacteria identification (GN ID) cards and antibiotic susceptibility testing (AST) N214 cards, was used for identification and antimicrobial susceptibility profiling of the bacterial isolates (BioMerièux, Marcy L’ É toile, France). Tested antibiotics included penicillin (ampicillin, piperacillin/tazobactam, and ampicillin-sulbactam), carbapenems (meropenem, ertapenem, and imipenem), fluoroquinolones (ciprofloxacin), tetracyclines (tetracycline), aminoglycosides (gentamicin), and trimethoprim/sulfamethoxazole. Results were interpreted according to the guidelines of the European Committee on Antimicrobial Susceptibility Testing (EUCAST guidelines, version 10.0, 2020; http://www.eucast.org/clinical_breakpoints/).

ESBL-producing bacteria were further confirmed using the combined double-disk synergy test with cefotaxime and ceftazidime alone or in combination with clavulanic acid (Becton, Dickinson and Company, Sparks, MD, USA) as described by the EUCAST guidelines version 9.0 (2019). In this study, ESBL-producing isolates of *E. coli* and *K. pneumoniae* were considered MDR if they were resistant to at least three classes of antibiotics. Quality control of each batch of the MacConkey agar containing 1 mg/L ceftazidime and 1 mg/L cefotaxime was performed using *E. coli* ATCC 25922 and a *bla*CTX-M positive *E. coli*.

### Polymerase chain reaction (PCR) and sequence detection of ESBL genotypes

For all confirmed ESBL-producing bacteria, a 10µL loopful of overnight pure colonies were transferred into saline. The solution was briefly vortexed, and the supernatant was discarded. The pellet was treated with 100 µL TE buffer (10:1) and heated at 95 °C for 5–10 min. The mixture was then centrifuged for 2 min. The supernatant containing the DNA was used for PCR analysis and sequencing. Molecular characterization of ESBL genes were performed by PCR for the presence of *bla*CTX-M (cefotaximase-Munich), *bla*TEM (Temoneira), and *bla*SHV (sulfhydryl variable enzyme) as described elsewhere [17, 18]. To differentiate *bla*CTX-M genes, previously designed specific target primers (Table 1) were used for PCR amplification [17]. The PCR product was then sent to Microsynth-Seqlab in Göttingen/Germany for sequencing. The resulting sequences were aligned and blasted using the Resfinder 4.1 software (https://cge.cbs.dtu.dk/services/ResFinder).

**Table 1:** Primers used for ESBL gene amplification by PCR

Target gene	Primer name	Sequences	Amplicon size (bp)
***bla*** **SHV**	SHV-F	5’-GCCGGGTTATTCTTATTTGTCCG-3’	1007
	SHV-R	5’-ATGCCGCCGCCAGTCA − 3’	
***bla*** **TEM**	TEM-F	5’-GTATCCGCTCATGAGACAATA-3’	966
	TEM-R	5’-TCTAAAGTATATATGAGTAAAC-3’	
***bla*** **CTX-M**	CTX-M-F	5’-TTTGCGATGTGCAGTACCAGTAA-3’	544
	CTX-M-R	5’-CGATATCGTTGGTGGTGCCATA-3’	
***bla*** **CTX-M-type 1**	CTX-M-1_F	5’-TCTTCCAGAATAAGGAATCCC-3’	909
	CTX-M-1_R	5’-CCGTTTCCGCTATTACAAAC-3’	
***bla*** **CTX-M-type 2**	CTX-M-2_F	5’-ATGATGACTCAGAGCATT-3’	884
	CTX-M-2_R	5’-TTATTGCATCAGAAACCGTG-3’	
***bla*** **CTX-M-type 8**	CTX-M-8_F	5’-TGATGAGACATCGCGTTAAG-3’	871
	CTX-M-8_R	5’-TAACCGTCGGTGACGATTTT-3’	
***bla*** **CTX-M-type 9**	CTX-M-9_F	5’-ATGGTGACAAAGAGARTGCAA-3’	873
	CTX-M-9_R	5’-CAGCCCTTCGGCGATGAT-3’	
***bla*** **CTX-M-type 14**	CTX-M-14_F	5’-ATTCAACAAAACCAGTTACAGCCC-3’	897
	CTX-M-14_R	5’-TTTGAGATGGTGACAAAGAGA-3’	

### Statistical analysis

Sociodemographic, clinical, and microbiological data collected from children enrolled in the study were entered and cleaned in Microsoft Excel. Statistical analyses were performed using the R language for statistical computing, version 4.0.2. All study variables were categorical and presented as frequencies with percentages. The Chi-squared test was used to compare count data against the null hypotheses. In cases where a cell had 5 values or less, Fisher’s exact test was applied.

## Results

### Characteristics of study participants

A total of 435 children were enrolled in the study. Of these, 47.1% (n/N = 205/435) presented with diarrhoea to the study hospital, and 52.8% (n/N = 230/435) were recruited among children without diarrhoea (Table 2). The number of female recruits (47.8%, n/N = 208/435) was slightly lower than that of male recruits (52.2%, n/N = 227/435). Diarrhoea samples were more common among males (58.5%, n/N = 120/205) than females (41.5%, n/N 85/205).

**Table 2:** Demographic characteristics of the study groups

Variable	Total (n = 435) (%)	Diarrhoea (n = 205) (%)	Non-Diarrhoea (n = 230) (%)	ESBL- Positive (n = 178) (%)	ESBL-EC (n = 163) (%)	ESBL-KP (n = 15) (%)
Age (months)						
0–12	149 (34.3)	87 (58.4)	62 (42)	55 (37)	48 (32.2)	7 (4.7)
13–24	117 (26.9)	67 (57.3)	50 (21.7)	45 (38.5)	42 (35.9)	3 (2.5)
25–36	83 (19.1)	38 (45.8)	45 (19.6)	37 (44.6)	34 (41)	3 (3.6)
37–48	46 (10.6)	10 (21.7)	36 (15.7)	19 (41.3)	17 (37)	2 (4.3)
49–60	40 (9.2)	3 (7.5)	37 (16.1)	22 (55)	22 (55)	0 (0)
**Sex**						
Male	227 (52.2)	120 (52.8)	107 (47.1)	90 (39.6)	82 (36.1)	8 (3.5)
Female	208 (47.8)	85 (40.8)	123 (59.1)	88 (42.3)	81 (38.9)	7 (3.3)

Table 2 shows the relationship between age, sex, and ESBL-producing isolates. ESBL-positive isolates were more common in children aged 49–60 months (55%, n/N = 22/40). The frequency was lowest among children between 0 and 12 months old. Age was not significantly associated with overall ESBL (*p* = 0.74), ESBL-EC (*p* = 0.52) or and ESBL-KP (*p* = 0.74) detection and ESBL-EC and ESBL-KP positivity were similarly distributed between male and female gender (*p* = 0.48).

Out of 435 children, 178 (40.9%) carried ESBL-producing isolates (ESBL-EC and ESBL-KP) (Table 3), and no difference in ESBL carriage between children with and without diarrhoea was observed (*p* = 0.75). In total, 187 ESBL-producing isolates (168 ESBL-EC and 19 ESBL-KP) were identified from 178 ESBL-positive children. Nine of the children had both ESBL-producing *E. coli* and *K. pneumoniae* (2.05%, n/N = 9/435). It was observed that almost all children without diarrhoea who were positive for ESBL bacteria (98.9%, n/N = 91/92) had ESBL-EC, whereas ESBL-EC was present in 89.5% (n/N = 77/86) of children with diarrhoea who were positive for ESBL bacteria. Almost all the ESBL-KP isolates (n = 13) were observed in children that had diarrhoea (86.6%, n/N = 13/15).

**Table 3:** ESBL-EC and ESBL-KP positive children in diarrhoea and diarrhoea group

	Total no. of Children (n = 435) (%)	Non-diarrhoea (n = 230) (%)	Diarrhoea (n = 205) (%)
ESBL			
Positive	178/435 (40.9)	92/230 (40.0)	86/205 (42.0)
*E. coli (alone)*	159/178 (89.3)	91/92 (98.9)	69/86 (80.2)
*K. pneumoniae (alone)*	10/178 (5.6)	1/92 (1.1)	9/86 (10.4)
*E. coli + K. pneumoniea*	9/178 (5)	1/92 (1.1)	8/86 (9.3)

ESBL: Extended Spectrum Beta Lactamase;

### Genotypic identification of ESBL genes among isolates

**Fig. 1:**
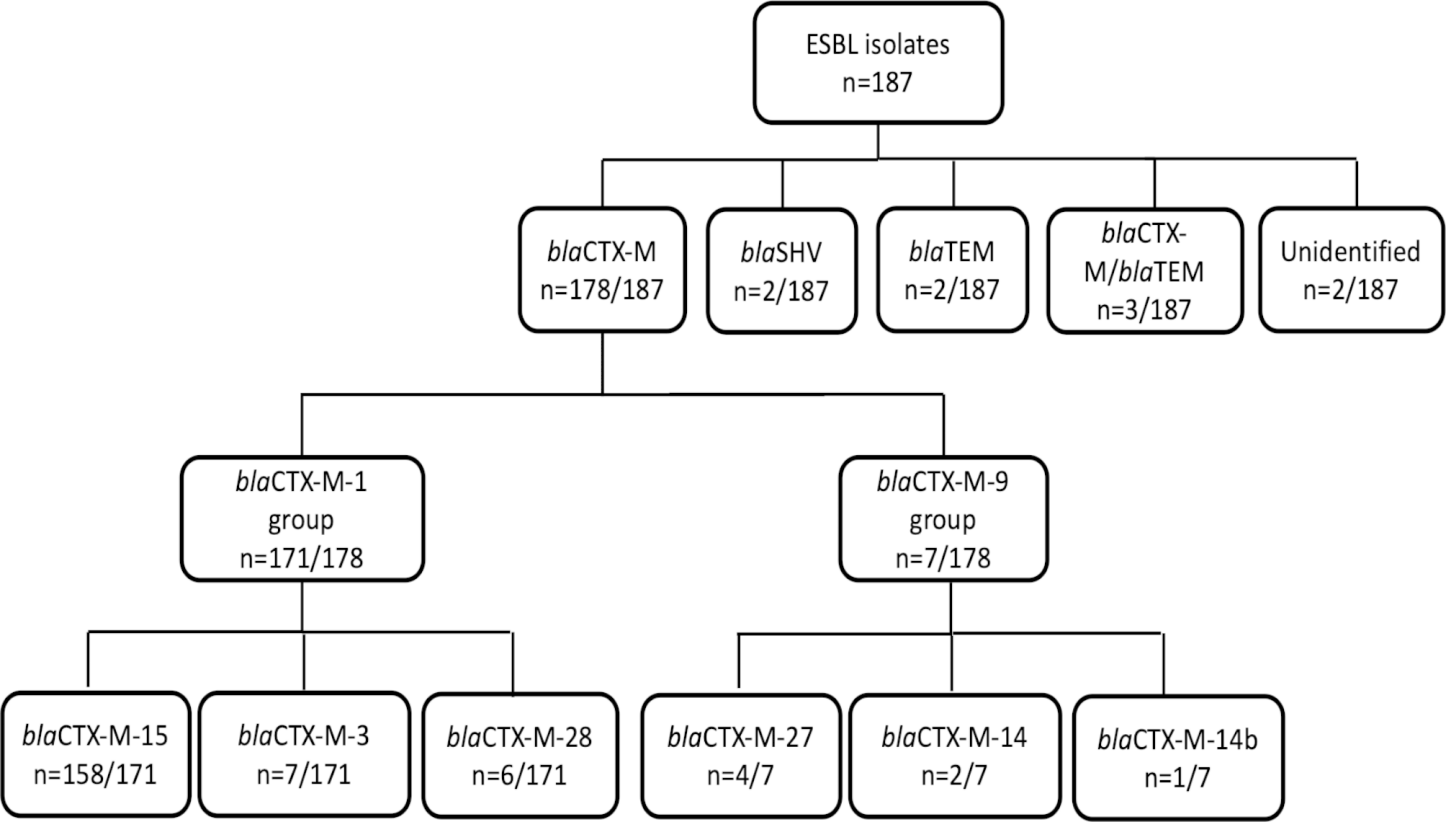
Distribution and frequency of different β-lactamase genes among the ESBL isolates

Figure 1 shows genotypic characterization identified three different beta-lactamase genes (*bla*CTX-M, *bla*SHV, and *bla*TEM) among 187 ESBL-EC and ESBL-KP isolates from 178 children. The majority were *bla*CTX-M positive (95.2%, n/N = 178/187), while a few carried *bla*CTX-M/*bla*TEM (1.6%, n/N = 3/187), *bla*SHV (1.1%, n/N = 2/187), or *bla*TEM (1.1%, n/N = 2/187). In two of the phenotypically confirmed ESBL isolates, none of the three genes (*bla*CTX-M, *bla*SHV, and *bla*TEM) were identified.

 Among isolates carrying the prevalent ESBL gene *bla*CTX-M, the majority of them were of group *bla*CTX-M-1 (96.1%, n/N = 171/178) (Fig. 1) whilst only seven were of group *bla*CTX-M-9 (3.9%, n/N = 7/178). Sequencing of the PCR product revealed *bla*CTX-M-15 (92.4%, n/N = 158/171) as the most common type of the *bla*CTX-M-1 group, and the remaining types were *bla*CTX-M-3 (4.1%, n/N = 7/171) and *bla*CTX-M-28 (3.5%, n/N = 6/171). Among the genes in group *bla*CTX-M-9, more than half were *bla*CTX-M-27 (57.1%, n/N = 4/7) while the remaining were *bla*CTX-M-14 (28.6%, n/N = 2/7) and *bla*CTX-M-14b (14.3%, n/N = 1/7).

 In terms of distribution of ESBL genes identified, *bla*CTX-M genes (n = 178) were equally distributed between the diarrhoea (50%, n/N = 89/178) and non-diarrhoea (50%, n/N = 89/178) stool types. *bla*TEM (n = 2) and *bla*SHV (n = 2) were rare and isolated from non-diarrhoea and diarrhoea stools, respectively. Among the *bla*CTX-M genes, *bla*CTX-M-1 was also distributed uniformly between the diarrhoea (52.0%, n/N = 89/171) and non-diarrhoea (48.0%, n/N = 82/171) samples whilst type *bla*CTX-M-9 was all isolated from non-diarrhoea stool samples (100.0%, n/N = 7/7).

 In terms of the distribution of beta-lactamase genes in ESBL-EC and ESBL-KP, *bla*CTX-M-15 were found to be the most common type in both bacterial species, with 85% (n/N = 143/168) and 79% (n/N = 15/19) abundance in ESBL-EC and ESBL-KP, respectively. The genes *bla*CTX-M-3, *bla*CTX-M-28, and *bla*SHV-12 was found in both organisms, whereas *bla*CTX-M-27, *bla*CTX-M-14 and 14b, and *bla*TEM were only found in ESBL-EC.

### Antimicrobial resistance of ESBL-EC and ESBL-KP isolates

**Fig. 2:**
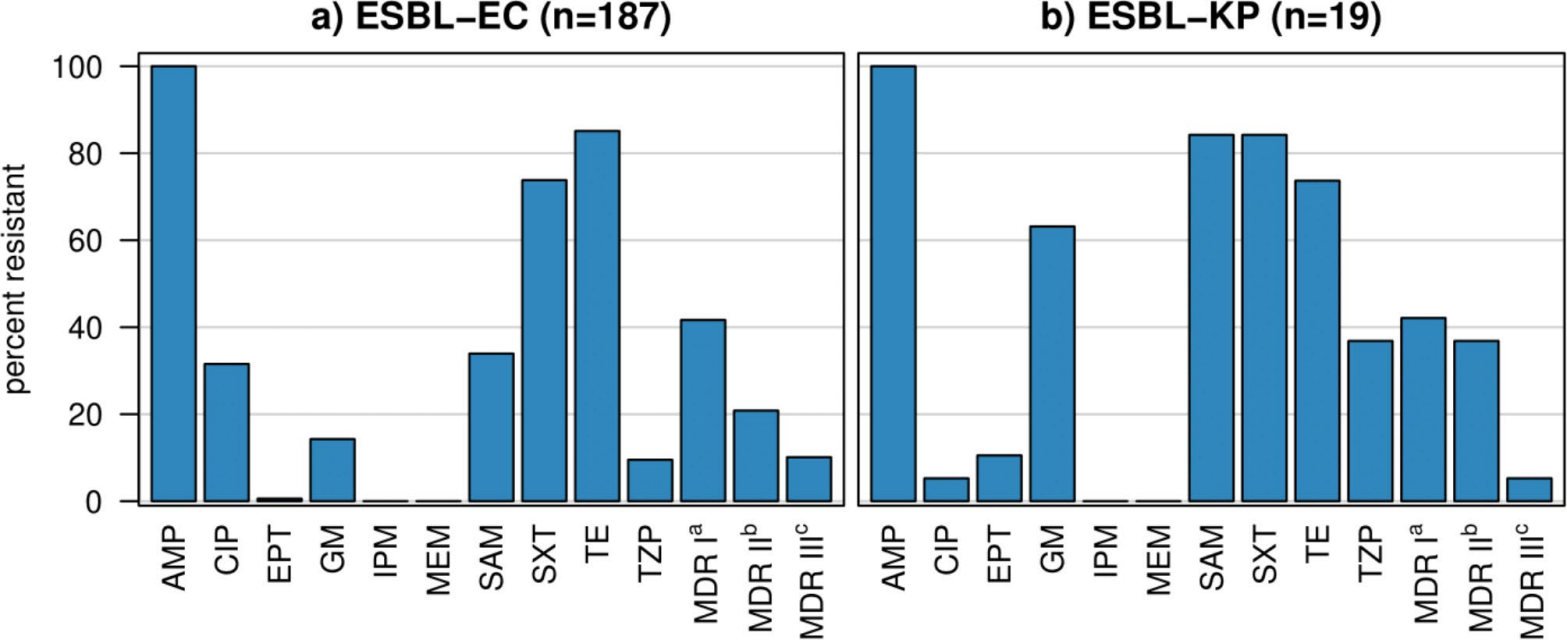
Antimicrobial resistance among ESBL-EC and ESBL-KP isolates from diarrhoea and non-diarrhoea stool samples of children. Abbreviations: Ampicillin (AMP), Ciprofloxacin (CIP), Ertapenem (ETP), Gentamicin (GM), Imipenem (IPM), Meropenem (MEM), Sulfamethoxazole-Trimethoprim (SXT), Tetracycline (TE), Piperacillin-Tazobactam (TZP), Ampicillin-Sulbactam (SAM). ^a^Multidrug resistance I defined as resistance to three antibiotics. ^b^Multidrug resistance II defined as resistance to four antibiotics. ^c^Multidrug resistance III defined as resistance to five antibiotics

ESBL-EC and ESBL-KP of this study showed resistance to antibiotics commonly used in Ghana (Fig. 2a and b). All isolates showed resistance to ampicillin. However, both ESBL-EC and ESBL-KP isolates were susceptible to meropenem and imipenem. Both ESBL-EC and ESBL-KP isolates showed the most resistance against tetracycline, with 85.1% (n/N = 143/168) and 73.6% (n/N = 14/19), followed by sulfamethoxazole-trimethoprim, against which the isolates showed 73.8% (n/N = 124/168) and 84.2% (n/N = 16/19) resistance, respectively. ESBL-EC isolates showed higher resistance against ciprofloxacin (31.5%, n/N = 53/168) compared to ESBL-KP isolates, which showed only 5.2% (n/N = 1/19) resistance. Whereas, ESBL-KP showed higher resistance towards ampicillin-sulbactam (84.2%, n/N = 16/19), gentamicin (63%, n/N = 12/19), and piperacillin-tazobactam (36.8%, n/N = 7/19) compared to ESBL-KP isolates, which showed only 34% (n/N = 57/168), 14.2% (n/N = 24/168), and 9.5% (n/N = 16/168) resistance, respectively. Notably, MDR was observed in 74% (n/N = 138/187) of the total isolates, with 41.7% (n/N = 78/187) being resistant to three classes of antibiotics, 22.4% (n/N = 42/187) being resistant to four classes of antibiotics, and 9.6% (n/N = 18/187) being resistant to five classes of antibiotics. MDR isolates were slightly higher in ESBL-KP (84.2%, n/N = 16/19) compared to ESBL-EC (78.5%, n/N = 132/168).

## Discussion

Carriage of ESBL-producing *Klebsiella pneumoniae* and *Escherichia coli* is one of the drivers of nosocomial and community infections, globally [7, 8]. Notwithstanding, it has received little attention in Ghana, particularly infections in children. This study explored ESBL-EC and ESBL-KP in children up to five years of age in Agogo, Ghana.

Our data shows that both children with and without diarrhoea are ESBL carriers, serving as a potential transmission reservoir. The overall prevalence of ESBL producers among the study population was 40.9% (Table 2). Out of which, 37.5% were ESBL-EC and only 3.4% were ESBL-KP. This finding is almost similar to the frequencies of ESBL producers among the adults from Ghana in the previous two studies, which reported frequencies of 41.5% and 49.3%, respectively [19, 20]. Interestingly, a previous study [14] carried out on young children in the same hospital as this study reported a much higher prevalence of ESBL-EC (61%) compared to the findings in this study. However, Falgenhauer et al. investigated hospitalized children in Agogo, who likely were more exposed to risk factors for carriage of ESBL, such as nosocomial carriage, antibiotics use, and longer hospitalization [21]. No age association with carriage of ESBL in children was found in our study (Table 2); however, a study in Tanzania reported high ESBL carriage in infants (0–3 months) compared to the older groups [15]. It could be due to mother-to-child transmission of the ESBL-producing isolates [22] but it may also reflect on antibiotic usage among children, especially for the management of diarrhoea [23].

Genomic characterization of the isolates from this study revealed *bla*CTX-M-15 as the most prevalent gene type in both diarrhoea and non-diarrhoea stool samples (Table 3), which is consistent with other studies [14, 15, 24] and subsequently confirms the current global state of the *bla*CTX-M-15 in community and hospital settings [25, 26]. This could be a consequence of the dissemination of group *bla*CTX-M-1, specifically *bla*CTX-M-15, which happens as a result of the mobilization of genetic platforms such as plasmids and transposons. It has been identified as the most dominant ESBL enzyme and is found in clinical isolates, community isolates, environmental isolates, and farm animals [14]. This is worrisome as the gut is the main reservoir for many Enterobacterales bacteria.

Remarkably, we found *bla*CTX-M-28 in both diarrhoea and non-diarrhoea stool samples, and to the best of our knowledge, this is the first time *bla*CTX-M-28 has been reported in Ghana. Unlike *bla*CTX-M-15, which has been frequently documented, *bla*CTX-M-28 has been sparingly described in SSA [27]. However, this gene has been reported in other parts of the world, such as Tunisia and Bosnia [28, 29]. *bla*CTX-M-28 may be underreported due to its close sequence similarity to *bla*CTX-M-15, which differs in only one nucleotide position [27]. The identification of *bla*CTX-M-28 in this study suggests either transmission of this gene from another country through movement of people [10] or a homoplastic mutation of that gene in an ancestor cell, leading to an identical sequence of *bla*CTX-M-28. We also identified *bla*CTX-M-27 in four of the ESBL-producing *E. coli* found in non-diarrhoea stool samples, indicating the diverse circulation of the *bla*CTX-M group in Agogo. The presence of other *bla*CTX-M groups besides *bla*CTX-M-15 emphasizes the dynamics and ongoing spread of ESBL genes in Agogo.

Due to the dissemination of these resistance genes, most developing countries are using broad-spectrum antibiotics as the clinical algorithm for managing patients with infection [30]. According to the latest guidelines from the Infectious Diseases Society of America, carbapenems, fluoroquinolones, and cotrimoxazole are recommended for managing most ESBL infections. Similarly, in Ghana, these are some of the common antibiotics used for treating ESBL infections; however, high resistance against fluoroquinolones and cotrimoxazole has already been reported [31, 32]. The antibiotic resistance profiles from our study also show high resistance to these antibiotics (Fig. 2). Among them, the most ineffective antibiotics were cotrimoxazole and tetracycline. This is of no surprise, as these two antibiotics have been used in Ghana for many years, and resistance against them has been reported in a wide range of bacteria [14, 33, 34]. The higher rates of resistance of ESBL-producers to cotrimoxazole, tetracycline, and ciprofloxacin are in accordance with other studies [12, 35], which could be because genes for resistance to these antibiotics are present on the same plasmid [12].

In contrast, all ESBL-EC and ESBL-KP were susceptible to the two carbapenems, meropenem and imipenem, which agrees with earlier studies in Nigeria and Tanzania [15, 36]. This might be due to the low or no usage of these antibiotics in Ghana [37].

We also observed a high prevalence of MDR (> 75%) for both ESBL-EC and ESBL-KP isolates in our study (Fig. 2), which is not surprising as ESBL-producing bacteria are frequently associated with co-resistance to other antimicrobial agents [14, 38]. This high rate of MDR could be a result of the extensive use of antibiotics in human medicine [39], in veterinary medicine [40] as well as other factors such as HGT and mobile genetic elements. Therefore, the ready availability of antibiotics in the local markets must be monitored. Most importantly, monitoring the resistance pattern exhibited by the MDR isolates and its impact on patient management is essential, especially in children.

In this study, sample size was not calculated prior to the study, and hence most of the calculations are exploratory and of a descriptive nature. Due to the low number of samples, e.g., for ESBL-KP, the results have to be interpreted with caution. Risk factors for the acquisition of the carriage of ESBL-producing *E. coli* and *K. pneumoniae* were not assessed. Data on antibiotic usage in children was not assessed in this study. Also, a test to ascertain whether the genes found were plasmid- or chromosomal-associated was not done. This is important in order to understand the impact of the resistance genes on the spread of AMR. Plasmid genes, for example, are mobile and easy to transfer to other bacteria and bacterial species.

## Conclusions

This study highlights the high frequency of stool carriage of ESBL-EC and ESBL-KP among children with or without diarrhoea in the Agogo community. Our study highlights the importance of this population as a possible reservoir and suggests that it may pose a risk for the transmission of drug resistance throughout the wider community. The study also found *bla*CTX-M-15 as the most prevalent gene in both diarrhoea and non-diarrhoea stools of children. It also reports for the first time the presence of *bla*CTX-M-28 in Ghana. The high frequency of ESBL genes found in this study is alarming, considering the limited diagnostic and treatment options that are available in resource-poor countries such as Ghana. The steady and continued increase of ESBL-producing bacteria and the associated antibiotic resistance has the potential to further select for even more resistant pathogens, such as carbapenem-resistant bacteria. We recommend the need for routine screening of ESBL-producing pathogens to optimize the use of antibiotics, and we encourage additional studies to evaluate these emerging genes and their risk factors in Ghana.

## Data Availability

All the data information analyzed during this study is available at KCCR.

## List of abbreviations

ESBL: Extended-spectrum beta-lactamase
ESBL-EC: ESBL-producing *Escherichia coli*
ESBL-KP: ESBL-producing *Klebsiella pneumoniae*
*bla*: beta-lactam
SHV: Sulfhydryl reagent variable
CTX-M: Cefotaxime-Munich
TEM: Temoneira
PCR: Polymerase chain reaction
AMR: Antimicrobial resistance
STEC: Shiga toxin-producing *E. coli*
EHEC: Enterohemorrhagic producing *E. coli*
SSA: sub-Saharan Africa
APH: Agogo Presbyterian Hospital
CWC: Child Welfare Clinic
KCCR: Kumasi Center for Collaborative Research in Tropical Medicine
GN ID: Gram-negative bacteria identification
AST: Antibiotic Susceptibility Testing
EUCAST: European Committee on Antimicrobial Susceptibility Testing
MDR: Multi-drug resistance

## References

[1] WHO. Global antimicrobial resistance and use surveillance system (GLASS) report [Internet]. WHO. 2020. Available from: http://www.who.int/glass/resources/publications/early-implementation-report-2020/en/.

[2] Murray CJ, Ikuta KS, Sharara F, Swetschinski L, Robles Aguilar G, Gray A et al. Global burden of bacterial antimicrobial resistance in 2019: a systematic analysis. Lancet [Internet]. 2022 Feb 12 [cited 2022 Jul 25];399(10325):629–55. Available from: http://www.thelancet.com/article/S0140673621027240/fulltext. 10.1016/S0140-6736(21)02724-0 PMC8841637 35065702

[3] Onduru OG, Mkakosya RS, Aboud S, Rumisha SF. Genetic Determinants of Resistance among ESBL-Producing Enterobacteriaceae in Community and Hospital Settings in East, Central, and Southern Africa: A Systematic Review and Meta-Analysis of Prevalence. Can J Infect Dis Med Microbiol = J Can des Mal Infect la Microbiol Médicale [Internet]. 2021 [cited 2022 Jul 25];2021. Available from: /pmc/articles/PMC8192179/. 10.1155/2021/5153237 PMC8192179 34122680

[4] Moyo P, Moyo E, Mangoya D, Mhango M, Mashe T, Imran M, et al. Prevention of antimicrobial resistance in sub-saharan Africa: what has worked? What still needs to be done? Journal of Infection and Public Health. Volume 16. Elsevier; 2023. pp. 632–9. 10.1016/j.jiph.2023.02.020 36870230

[5] Li Q, Chang W, Zhang H, Hu D, Wang X. The role of plasmids in the multiple antibiotic resistance transfer in ESBLs-producing *Escherichia coli* isolated from wastewater treatment plants. Front Microbiol. 2019;10(APR):633. 10.3389/fmicb.2019.00633 PMC6456708 31001218

[6] Chong Y, Shimoda S, Shimono N. Current epidemiology, genetic evolution and clinical impact of extended-spectrum β-lactamase-producing *Escherichia coli* and *Klebsiella pneumoniae*. Infect Genet Evol. 2018 Jul;1:61:185–8. 10.1016/j.meegid.2018.04.005 29626676

[7] Obeng-Nkrumah N, Labi A-K, Addison NO, Labi JEM, Awuah-Mensah G (2016). Trends in paediatric and adult bloodstream infections at a ghanaian referral hospital: a retrospective study. Ann Clin Microbiol Antimicrob.

[8] Silva Y, Ferrari R, Marin VA, Adam C, Junior C. REVIEW ARTICLE A global overview of β-lactam resistance genes in *Klebsiella pneumonia* H. 2019;22–34.

[9] Khairy RMM, Fathy ZA, Mahrous DM, Mohamed ES, Abdelrahim SS. Prevalence, phylogeny, and antimicrobial resistance of *Escherichia coli* pathotypes isolated from children less than 5 years old with community acquired- diarrhea in Upper Egypt. BMC Infect Dis [Internet]. 2020 Dec 1 [cited 2022 Jun 27];20(1). Available from: /pmc/articles/PMC7708180/. 10.1186/s12879-020-05664-6 PMC7708180 33256619

[10] Ouchar Mahamat O, Tidjani A, Lounnas M, Hide M, Benavides J, Somasse C et al. Fecal carriage of extended-spectrum β-lactamase-producing Enterobacteriaceae in hospital and community settings in Chad. Antimicrob Resist Infect Control [Internet]. 2019 Oct 31 [cited 2021 Aug 16];8(1):1–7. Available from: https://aricjournal.biomedcentral.com/articles/10.1186/s13756-019-0626-z. 10.1186/s13756-019-0626-z PMC6824111 31695911

[11] Berendes D, Kirby A, Brown J, Wester AL. Human faeces-associated extended-spectrum β-lactamase-producing *Escherichia coli* discharge into sanitation systems in 2015 and 2030: a global and regional analysis. Lancet Planet Heal. 2020 Jun 1;4(6):e246–55. 10.1016/S2542-5196(20)30099-1 PMC10906806 32559441

[12] Sapkota B, Yadav SK, Dhungana G, Ansari S, Mishra SK. Intestinal Carriage of Extended-Spectrum β-Lactamase- (ESBL-) Possessing *Escherichia coli* and *Klebsiella* Species among Nepalese Health Science and Non-Health Science Students. Can J Infect Dis Med Microbiol = J Can des Mal Infect la Microbiol Médicale [Internet]. 2021 [cited 2022 Jul 25];2021. Available from: /pmc/articles/PMC8052163/. 10.1155/2021/4767429 PMC8052163 33897921

[13] Alonso CA, Zarazaga M, Ben Sallem R, Jouini A, Ben Slama K, Torres C. Antibiotic resistance in *Escherichia coli* in husbandry animals. The african perspective. Lett Appl Microbiol. 2017 Feb. 10.1111/lam.12724 28208218

[14] Falgenhauer L, Imirzalioglu C, Oppong K, Akenten CW, Hogan B, Krumkamp R et al. Detection and characterization of ESBL-producing *Escherichia coli* from humans and poultry in Ghana. Front Microbiol [Internet]. 2019;10(JAN). Available from: www.frontiersin.org. 10.3389/fmicb.2018.03358 PMC6340976 30697208

[15] Tellevik MG, Blomberg B, Kommedal Ø, Maselle SY, Langeland N, Moyo SJ (2016). High prevalence of faecal carriage of esbl-producing Enterobacteriaceae among children in Dar es Salaam, Tanzania. PLoS ONE.

[16] Sapkota B, Yadav SK, Dhungana G, Ansari S, Mishra SK. Intestinal Carriage of Extended-Spectrum β-Lactamase- (ESBL-) Possessing *Escherichia coli* and Klebsiella Species among Nepalese Health Science and Non-Health Science Students. Can J Infect Dis Med Microbiol = J Can des Mal Infect la Microbiol Médicale [Internet]. 2021 [cited 2023 Feb 11];2021. Available from: /pmc/articles/PMC8052163/. 10.1155/2021/4767429 PMC8052163 33897921

[17] Belmar Campos C, Fenner I, Wiese N, Lensing C, Christner M, Rohde H et al. Prevalence and genotypes of extended spectrum beta-lactamases in Enterobacteriaceae isolated from human stool and chicken meat in Hamburg, Germany. Int J Med Microbiol. 2014. 10.1016/j.ijmm.2014.04.012 24856867

[18] McGettigan SE, Hu B, Andreacchio K, Nachamkin I, Edelstein PH. Prevalence of CTX-M β-lactamases in Philadelphia, Pennsylvania. J Clin Microbiol. 2009. 10.1128/JCM.00319-09 PMC2738063 19587301

[19] Deku JG, Duedu KO, Ativi E, Kpene GE, Feglo PK (2021). Occurrence and distribution of extended-spectrum β-lactamase in clinical *Escherichia coli* isolates at Ho Teaching Hospital in Ghana. Ghana Med J [Internet].

[20] Obeng-Nkrumah N, Hansen DS, Awuah-Mensah G, Blankson NK, Frimodt-Møller N, Newman MJ, Opintan JA, Krogfelt KA. High level of colonization with third-generation cephalosporin-resistant Enterobacterales in African community settings, Ghana. 10.1016/j.diagmicrobio.2023.115918 37058979

[21] Kibwana UO, Majigo M, Kamori D, Manyahi J. High fecal carriage of extended Beta Lactamase producing Enterobacteriaceae among adult patients admitted in referral hospitals in Dar es Salaam, Tanzania. BMC Infect Dis [Internet]. 2020 Jul 31 [cited 2022 Jun 2];20(1). Available from: /pmc/articles/PMC7393831/. 10.1186/s12879-020-05272-4 PMC7393831 32736605

[22] Danino D, Melamed R, Sterer B, Porat N, Hazan G, Gushanski A et al. Mother-to-child transmission of extended-spectrum-beta-lactamase-producing Enterobacteriaceae. J Hosp Infect [Internet]. 2018 Sep 1 [cited 2022 Jul 25];100(1):40–6. Available from: http://www.journalofhospitalinfection.com/article/S0195670118300367/fulltext. 10.1016/j.jhin.2017.12.024 29330015

[23] El-Khoury M, Banke K, Sloane P. Improved childhood diarrhea treatment practices in Ghana: a pre-post evaluation of a comprehensive private-sector program. Glob Heal Sci Pract. 2016. 10.9745/GHSP-D-16-00021 PMC4982250 27353619

[24] Birgy A, Cohen R, Levy C, Bidet P, Courroux C, Benani M et al. Community faecal carriage of extended-spectrum beta-lactamase-producing Enterobacteriaceae in french children. BMC Infect Dis 2012 121 [Internet]. 2012 Nov 21 [cited 2021 Jul 23];12(1):1–5. Available from: https://bmcinfectdis.biomedcentral.com/articles/10.1186/1471-2334-12-315. 10.1186/1471-2334-12-315 PMC3579697 23171127

[25] Ur Rahman S, Ali T, Ali I, Khan NA, Han B, Gao J. The Growing Genetic and Functional Diversity of Extended Spectrum Beta-Lactamases. Biomed Res Int. 2018;2018. 10.1155/2018/9519718 PMC5892270 29780833

[26] Barrios H, Garza-Ramos U, Mejia-Miranda I, Reyna-Flores F, Sánchez-Pérez A, Mosqueda-García D, et al. ESBL-producing *Escherichia coli* and *Klebsiella pneumoniae*: the most prevalent clinical isolates obtained between 2005 and 2012 in Mexico. J Glob Antimicrob Resist. 2017 Sep;1:10:243–6. 10.1016/j.jgar.2017.06.008 28739224

[27] Alfaresi M, Kim Sing G, Senok A. First Report of bla CTX-M-28 in Enterobacteriaceae Isolates in the United Arab Emirates. J Pathog [Internet]. 2018;2018:1–5. Available from: 10.1155/2018/1304793. 10.1155/2018/1304793 PMC5822763 29593911

[28] Ibrahimagić A, Bedenić B, Kamberović F, Uzunović S (2015). High prevalence of CTX-M-15 and first report of CTX-M-3, CTX-M-22, CTX-M-28 and plasmid-mediated AmpC beta-lactamase producing Enterobacteriaceae causing urinary tract infections in Bosnia and Herzegovina in hospital and community settings. J Infect Chemother.

[29] Ben Achour N, Mercuri PS, Power P, Belhadj C, Ben Moussa M, Galleni M, et al. First detection of CTX-M-28 in a tunisian hospital from a cefotaxime-resistant *Klebsiella pneumoniae* strain. Pathol Biol. 2009 Jul;57(1):343–8. 10.1016/j.patbio.2008.07.016 18834674

[30] Dodoo CC, Orman E, Alalbila T, Mensah A, Jato J, Mfoafo KA et al. Antimicrobial prescription pattern in ho teaching hospital, ghana: Seasonal determination using a point prevalence survey. Antibiotics [Internet]. 2021 Feb 18 [cited 2021 Aug 17];10(2):1–11. Available from: https://www.mdpi.com/2079-6382/10/2/199/htm. 10.3390/antibiotics10020199 PMC7923162 33670731

[31] Asamoah B, Labi AK, Gupte HA, Davtyan H, Peprah GM, Adu-Gyan F et al. High Resistance to Antibiotics Recommended in Standard Treatment Guidelines in Ghana: A Cross-Sectional Study of Antimicrobial Resistance Patterns in Patients with Urinary Tract Infections between 2017–2021. Int J Environ Res Public Health [Internet]. 2022 Dec 1 [cited 2023 Apr 27];19(24):16556. Available from: https://www.mdpi.com/1660-4601/19/24/16556/htm. 10.3390/ijerph192416556 PMC9779193 36554436

[32] Tamma PD, Aitken SL, Bonomo RA, Mathers AJ, van Duin D, Clancy CJ. Infectious Diseases Society of America 2022 Guidance on the Treatment of Extended-Spectrum β-lactamase Producing Enterobacterales (ESBL-E), Carbapenem-Resistant Enterobacterales (CRE), and P*seudomonas aeruginosa* with Difficult-to-Treat Resistance (DTR-P. aeruginosa). Clin Infect Dis [Internet]. 2022 Aug 25 [cited 2023 Apr 27];75(2):187–212. Available from: https://academic.oup.com/cid/article/75/2/187/6570801. 10.1093/cid/ciac268 PMC9890506 35439291

[33] Duedu KO, Offei G, Codjoe FS, Donkor ES. Multidrug Resistant Enteric Bacterial Pathogens in a Psychiatric Hospital in Ghana: Implications for Control of Nosocomial Infections. Int J Microbiol. 2017;2017. 10.1155/2017/9509087 PMC5606046 29038662

[34] Labi AK, Obeng-Nkrumah N, Addison NO, Donkor ES. Salmonella blood stream infections in a tertiary care setting in Ghana. BMC Infect Dis [Internet]. 2014 Dec 21 [cited 2022 Jul 25];14(1):1–10. Available from: https://bmcinfectdis.biomedcentral.com/articles/10.1186/s12879-014-0697-7. 10.1186/s12879-014-0697-7 PMC4297363 25528352

[35] Subramanya SH, Bairy I, Metok Y, Baral BP, Gautam D, Nayak N. Detection and characterization of ESBL-producing Enterobacteriaceae from the gut of subsistence farmers, their livestock, and the surrounding environment in rural Nepal. Sci Rep [Internet]. 2021;11(1):2091. Available from: 10.1038/s41598-021-81315-3. 10.1038/s41598-021-81315-3 PMC7822894 33483551

[36] Saka HK, García-Soto S, Dabo NT, Lopez-Chavarrias V, Muhammad B, Ugarte-Ruiz M et al. Molecular detection of extended spectrum β-lactamase genes in *Escherichia coli* clinical isolates from diarrhoeic children in Kano, Nigeria. PLoS One [Internet]. 2020;15(12 December). Available from: 10.1371/journal.pone.0243130. 10.1371/journal.pone.0243130 PMC7714196 33270734

[37] Labi AK, Obeng-Nkrumah N, Sunkwa-Mills G, Bediako-Bowan A, Akufo C, Bjerrum S et al. Antibiotic prescribing in paediatric inpatients in Ghana: A multi-centre point prevalence survey [Internet]. Vol. 18, BMC Pediatrics. BioMed Central Ltd.; 2018 [cited 2022 Jan 5]. p. 1–9. Available from: https://bmcpediatr.biomedcentral.com/articles/10.1186/s12887-018-1367-5. 10.1186/s12887-018-1367-5 PMC6302438 30572851

[38] Tola MA, Abera NA, Gebeyehu YM, Dinku SF, Tullu KD. High prevalence of extended-spectrum betalactamase- producing *Escherichia coli* and *Klebsiella pneumoniae* fecal carriage among children under five years in Addis Ababa, Ethiopia. PLoS One [Internet]. 2021;16(10 October):1–16. Available from: 10.1371/journal.pone.0258117. 10.1371/journal.pone.0258117 PMC8486131 34597328

[39] Yevutsey SK, Buabeng KO, Aikins M, Anto BP, Biritwum RB, Frimodt-Møller N et al. Situational analysis of antibiotic use and resistance in Ghana: policy and regulation. BMC Public Health. 2017;17(1). 10.1186/s12889-017-4910-7 PMC5701378 29169340

[40] Adeapena W, Afari-Asiedu S, Najjemba R, Griensven J, Van, Delamou A, Ohene Buabeng K (2021). Antibiotic use in a municipal Veterinary Clinic in Ghana. Trop Med Infect Dis.

